# Expression profiles of interferon-related genes in cells infected with influenza A viruses or transiently transfected with plasmids encoding viral RNA polymerase

**DOI:** 10.3906/biy-2005-73

**Published:** 2021-02-09

**Authors:** Elif ÇAĞLAYAN, Kadir TURAN

**Affiliations:** 1 Enstitute of Health Sciences, Marmara University, İstanbul Turkey; 2 Department of Basic Pharmaceutical Sciences, Faculty of Pharmacy, Marmara University, İstanbul Turkey

**Keywords:** Influenza A viruses, interferon response, PCR array, influenza RdRP

## Abstract

Influenza A viruses frequently change their genetic characteristics, which leads to the emergence of new viruses. Consequently, elucidation of the relationship between influenza A virus and host cells has a great importance to cope with viral infections. In this study, it was aimed to determine expression profiles of interferon response genes in human embryonic kidney 293 (HEK293) cells infected with human (A/WSN-H1N1) and avian influenza A viruses (duck/Pennsylvania/10218/84/H5N2) or transfected with plasmids encoding viral RdRP subunits and, to obtain clues about the genes that may be important for the viral pathogenesis. The HEK293 cells cultured in a 12-well plate were infected with influenza A viruses or transfected with plasmids encoding viral polymerase. Total RNA extraction and cDNA preparation were carried out with commercial kits. Qiagen 96-well-RT^2^ Profiler PCR Array plates designated for interferons response genes were used for quantitation of the transcripts. The relative quantities of transcripts were normalized with STAT3 gen, and the results were evaluated. Quantitative RT-PCR results showed that there are substantial differences of the interferon response gene transcription in cells infected with viruses or transfected with plasmids. A higher number of interferon-related genes were found to be downregulated in the cells infected with DkPen compared to WSN. On the other hand, significant differences in the expression profiles of interferon response genes were observed in the cells expressing viral PA protein. In particular, avian influenza PA protein was found to cause more aggressive changes on the transcript levels. Human and avian influenza A viruses cause a substantial change in interferon response gene expression in HEK293 cells. However, a higher number of genes were downregulated in the cells infected with avian influenza DkPen compared to WSN. It has been also concluded that the viral PA protein is one of the important viral factors affecting the transcript level of host genes.

## 1. Introduction

Influenza A viruses are enveloped viruses classified in the *Orthomyxoviridae* family. The viral genome consists of eight single-strand RNA molecules having negative polarity (u1da0). These viruses infect a wider range of host species than other types of RNA viruses. Although the natural reservoirs of influenza A viruses are aquatic bird species, they also infect humans, pigs, horses, and many marine mammal species. The main reasons influenza A viruses cause such widespread infections are the high mutation frequency and the reassortment of gene segments during coinfections (u1da1). New virus subtypes are emerging as a result of genome mutations causing changes in the amino acid composition of viral proteins, primarily the virus’s surface antigen proteins, hemagglutinin (HA) and neuraminidase (NA). Influenza A viruses are divided into several subtypes based on their surface glycoproteins, i.e. HA and NA. So far, 18 HA (H1 to H18) and 11 NA (N1 to N11) types, which differ antigenically, have been identified (u1da2). These mutations give the viruses high adaptability and easy transmission ability. Consequently, influenza A viruses can cause flu outbreaks in humans with high rates of morbidity and mortality worldwide and are regarded as a major global health threat. Influenza A virus infections sometimes appear as mild respiratory tract diseases but at others cause severe respiratory failure and can result in death (u1da3). The host’s cellular defense mechanism plays an important role in the emergence of infections with different severities in addition to elements arising from the structural properties of the viruses. The structure of influenza A virus surface antigens (HA and NA), the receptors to which HA protein binds, and intracellular defense mechanisms of the host cells are important factors that determine host specificity (u1da4; u1da5; u1da6). On the other hand, the studies have revealed that protein subunits of the viral RNA-dependent RNA polymerase (RdRP) enzyme have a great importance in terms of viral pathogenicity (u1da7). Reverse genetic studies show that the presence of lysine (K) at 627 positions of the PB2 protein of IAV H5N1 human isolate causes high pathogenicity in mice, while the presence of Glu (E) causes mild symptoms (u1da8). K577E mutation in PB1 protein, another subunit of the viral RdRP, is also known to provide adaptation of the H9N2 virus to mammalian cells (u1da9). In a study carried out with influenza A virus minireplicons, it was revealed that the RdRP of avian type influenza A/duck/Pennsylvania/10218/84 (DkPen) has very low activity compared to enzymes of the human type A/WSN/33 (WSN), and the N-terminal endonuclease domain of the PA subunit of the RdRP is essential for mammalian adaptation (u1daa). Another viral protein, the nonstructural NS1, has also been shown to be important for the adaptation of avian influenza A H9N2 subtype (A/Ck/Korea/163/04H9N2) to mammalian cells (u1dab)

The virus-host specificity and pathogenicity do not depend only on viral factors, as the host’s intracellular defense mechanisms also play an important role. Immediately after a viral infection, the innate immune response of the cells is critical for defense against the viruses. Consequently, the elucidation of complex interactions between the host and the viruses is the most important part of research. While the viral infection causes activation of the host’s interferon system, the virus activates mechanisms to cope with this defense system. Interferon response-related proteins such as 2’-5’-oligoadenylate synthetase (OAS), protein kinase R (PKR) and Mx proteins, which are stimulated by interferons in the cells, are also very important factors affecting the course of influenza virus replication (u1dac; u1dad). The PKR enzyme phosphorylates eukaryotic initiation factor 2 (eIF-2) is activated in response to new viral infections while phosphorylated eIF-2 forms an inactive complex with a protein called eIF2B to reduce protein synthesis within the cells (u1dae). Another cellular enzyme induced with interferons, RNAse L, breaks down the RNAs in the cells to further reduce both viral and host protein synthesis. Inhibition of protein synthesis in the cells blocks virus replication and damages the infected host cells (u1daf). The techniques for analysis of multiple gene expression profiles are used to outline complex interactions such as virus-host cell relationships. Among these techniques, microarray analysis is especially useful in determining the simultaneous expression profiles of thousands of genes (u1db0). The PCR-based array technique, which is another system, does not allow the analysis of as many genes as microarray techniques but gives much more sensitive results. Consequently, in this study, we analyzed the expression profiles of a limited number of genes associated with interferon response in human embryonic kidney 293 (HEK293) cells infected with influenza A viruses or transfected with plasmid DNAs encoding viral RdRP subunits. The results reveal that not only the type of virus used in infection but also the viral polymerase enzyme subunits synthesized in the cells by transient transfection cause significant differences in the expression profiles of the genes affected by interferons. In particular, influenza DkPen viruses were found to negatively affect the gene transcript levels in the host cells.

## 2. Material and methods

### 2.1. Cells

HEK293 cells were used for viral infection and/or transient transfection experiments. Madin-Darby canine kidney (MDCK) cells were used for virus propagation and titrations (u1db1). The cells were cultured in Dulbecco’s modified eagle medium (DMEM) supplemented with 10% heat-inactivated fetal calf serum (Gibco Laboratories, Gaithersburg, MD, USA), 100 IU/mL penicillin, 100 µg/mL streptomycin, 2 mM glutamine, and 1.5 mg/mL sodium bicarbonate at 37 °C in a humidified incubator with 5% CO2.

### 2.2. Viruses

Human influenza A/WSN/33/H1N1 (WSN) and low pathogenic avian influenza A/duck/Pennsylvania/10,218/84/H5N2 (DkPen) viruses were used. The viruses were obtained from Laboratory of Infection Biology (Virology), Graduate School of Comprehensive Human Sciences (University of Tsukuba/Japan) and propagated in specific pathogen-free (SPF) embryonated chicken eggs and/or MDCK cells. The viral titer was defined using a standard plaque assay (u1db2).

### 2.3. Plasmid vectors

The expression plasmids constructed in previous studies (u1db3; u1db4) were used for the transient synthesis of influenza A virus RdRP subunits (PB2 + PB1 + PA) or only PA protein in transfected HEK293 cells. To determine the effects of the influenza A virus RNA polymerase subunit PA protein in transcription and/or translation in HEK293 cells, the pSEAP (Clontech) reporter plasmid and the pCHA-ACTB plasmid (u1db5) encoding HA-tagged human beta-actin protein were used. The plasmid vectors coding chimeric PA proteins (consisting of amino-terminal moiety of WSN PA and carboxyl-terminal moiety of DkPen PA, or vice versa) were constructed with subcloning using pCAGGS-PA(W) and pCAGGS-PA(D) plasmids (u1db7). For this purpose, plasmid DNA was first digested with BsmI restriction enzyme (New England Biolabs Ltd., Hitchin, UK) via a cut site in the vector and then DraIII (New England Biolabs Ltd.) enzyme was used. The DNA fragments were purified with an agarose gel extraction kit (QiaexII, Qiagen GmbH, Hilden, Germany). The fragments coding the carboxyl terminal half of the PA proteins and the remaining part of the plasmids were cross-ligated with the T4-DNA ligase (Takara Bio Inc., Shiga, Japan). The resulting plasmids were named pCAGGS-PA(W-D) and pCAGGS-PA(D-W). The plasmid vectors encoding the amino-terminal (nPA) or carboxyl-terminal (cPA) moiety of influenza PA proteins were obtained with inverse PCR using specific phosphorylated primers and the pCAGGS-PA(D) and pCAGGS-PA(W) plasmids as templates. To amplify pCAGGS-nPA(D) and pCAGGS-cPA(D) by inverse PCR via the pCAGGS-PA(D) template; “5´-AGATCTTTTTCCCTCTGCCAAAAATTATG-3´ (Vect-For) / 5´-CTAGTTCTTTGTCTTTGGGATCTTC-3´ (nPA-D-Rev)” and “5´-ATGAAGAAAACAAGCCAATTGAAG-3´ (cPA-D-For) / 5´-GGCGGCGCGAGCTCGAGG-3´ (Vect-Rev)” primer pairs were used, respectively. Similarly, pCAGGS-nPA(W) and pCAGGS-cPA(W) DNA molecules were amplified via the pCAGGS-PA(W) template with inverse PCR using “Vect-For / 5´ - CTAATTTTTAGTCCTTGGAATTTTCTC - 3´ (nPA-W-Rev)” primer pairs and “5´ - ATGAAGAAAACGAGTCAGTTAAAG - 3´ (cPA-W-For) / Vect-Rev” primer pairs, respectively. The PCR products were purified with a gel extraction kit and self-ligated with T4 DNA ligase. All plasmids were checked by DNA sequencing.

### 2.4. PCR-array panels

The 96-well “RT² Profiler PCR Array” (PAHS-016ZD-6) plates developed by Qiagen (Qiagen GmbH) compatible with the “Bio-Rad CFX96” thermal cycler was used. The plates included interferon/interferon receptor genes or interferon-induced genes (84 different genes), five housekeeping control genes, three positive controls, three reverse transcription controls and one genomic DNA contamination control (Table 1).

**Table 1:** The interferon-related genes quantitated in HEK293 cells by PCR array techniques.

Symbol	:	GenBank	Description	Symbol	:	GenBank	Description
ACTB	:	NM_001101	Actin, beta	IL10	:	NM_000572	Interleukin 10
ADAR	:	NM_001111	Adenosine deaminase, RNA-specific	IL15	:	NM_000585	Interleukin 15
B2M	:	NM_004048	Beta-2-microglobulin	IL6	:	NM_000600	Interleukin 6 (interferon, beta 2)
BST2	:	NM_004335	Bone marrow stromal cell antigen 2	IRF1	:	NM_002198	Interferon regulatory factor 1
CASP1	:	NM_033292	Caspase 1, apoptosis-related cysteine peptidase	IRF2	:	NM_002199	Interferon regulatory factor 2
CAV1	:	NM_001753	Caveolin 1, caveolae protein, 22kDa	IRF3	:	NM_001571	Interferon regulatory factor 3
CCL2	:	NM_002982	Chemokine (C-C motif) ligand 2	IRF5	:	NM_001098629	Interferon regulatory factor 5
CCL5	:	NM_002985	Chemokine (C-C motif) ligand 5	IRF7	:	NM_001572	Interferon regulatory factor 7
CD70	:	NM_001252	CD70 molecule	IRF9	:	NM_006084	Interferon regulatory factor 9
CD80	:	NM_005191	CD80 molecule	ISG15	:	NM_005101	ISG15 ubiquitin-like modifier
CD86	:	NM_006889	CD86 molecule	ISG20	:	NM_002201	Interferon stimulated exonuclease gene 20kDa
CDKN1B	:	NM_004064	Cyclin-dependent kinase inhibitor 1B (p27, Kip1)	JAK1	:	NM_002227	Janus kinase 1
CIITA	:	NM_000246	Class II, major histocompatibility complex, transactivator	JAK2	:	NM_004972	Janus kinase 2
CRP	:	NM_000567	C-reactive protein, pentraxin-related	MAL	:	NM_002371	Mal, T-cell differentiation protein
CXCL10	:	NM_001565	Chemokine (C-X-C motif) ligand 10	MET	:	NM_000245	Met proto-oncogene (hepatocyte growth factor receptor)
DDX58	:	NM_014314	DEAD (Asp-Glu-Ala-Asp) box polypeptide 58	MNDA	:	NM_002432	Myeloid cell nuclear differentiation antigen
EIF2AK2	:	NM_002759	Eukaryotic translation initiation factor 2-alpha kinase 2	MX1	:	NM_002462	Myxovirus (influenza virus) resistance 1, interferon-inducible protein p78
GAPDH	:	NM_002046	Glyceraldehyde-3-phosphate dehydrogenase	MX2	:	NM_002463	Myxovirus (influenza virus) resistance 2
GBP1	:	NM_002053	Guanylate binding protein 1, interferon-inducible	MYD88	:	NM_002468	Myeloid differentiation primary response gene (88)
HLA-A	:	NM_002116	Major histocompatibility complex, class I, A	NMI	:	NM_004688	N-myc (and STAT) interactor
HLA-B	:	NM_005514	Major histocompatibility complex, class I, B	NOS2	:	NM_000625	Nitric oxide synthase 2, inducible
HLA-E	:	NM_005516	Major histocompatibility complex, class I, E	OAS1	:	NM_002534	2’-5’-oligoadenylate synthetase 1, 40/46kDa
HLA-G	:	NM_002127	Major histocompatibility complex, class I, G	OAS2	:	NM_002535	2’-5’-oligoadenylate synthetase 2, 69/71kDa
HPRT1	:	NM_000194	Hypoxanthine phosphoribosyltransferase 1	PML	:	NM_033238	Promyelocytic leukemia
IFI16	:	NM_005531	Interferon, gamma-inducible protein 16	PRKCZ	:	NM_002744	Protein kinase C, zeta
IFI27	:	NM_005532	Interferon, alpha-inducible protein 27	PSME2	:	NM_002818	Proteasome (prosome, macropain) activator subunit 2 (PA28 beta)
IFI30	:	NM_006332	Interferon, gamma-inducible protein 30	RPLP0	:	NM_001002	Ribosomal protein, large, P0
IFI6	:	NM_002038	Interferon, alpha-inducible protein 6	SHB	:	NM_003028	Src homology 2 domain containing adaptor protein B
IFIH1	:	NM_022168	Interferon induced with helicase C domain 1	SOCS1	:	NM_003745	Suppressor of cytokine signaling 1
IFIT1	:	NM_001548	Interferon-induced protein with tetratricopeptide repeats 1	STAT1	:	NM_007315	Signal transducer and activator of transcription 1, 91kDa
IFIT2	:	NM_001547	Interferon-induced protein with tetratricopeptide repeats 2	STAT2	:	NM_005419	Signal transducer and activator of transcription 2, 113kDa
IFIT3	:	NM_001549	Interferon-induced protein with tetratricopeptide repeats 3	STAT3	:	NM_003150	Signal transducer and activator of transcription 3
IFITM1	:	NM_003641	Interferon induced transmembrane protein 1 (9-27)	TAP1	:	NM_000593	Transporter 1, ATP-binding cassette, sub-family B (MDR/TAP)
IFITM2	:	NM_006435	Interferon induced transmembrane protein 2 (1-8D)	TICAM1	:	NM_182919	Toll-like receptor adaptor molecule 1
IFITM3	:	NM_021034	Interferon induced transmembrane protein 3	TIMP1	:	NM_003254	TIMP metallopeptidase inhibitor 1
IFNA1	:	NM_024013	Interferon, alpha 1	TLR3	:	NM_003265	Toll-like receptor 3
IFNA2	:	NM_000605	Interferon, alpha 2	TLR7	:	NM_016562	Toll-like receptor 7
IFNA4	:	NM_021068	Interferon, alpha 4	TLR8	:	NM_138636	Toll-like receptor 8
IFNAR1	:	NM_000629	Interferon (alpha, beta and omega) receptor 1	TLR9	:	NM_017442	Toll-like receptor 9
IFNAR2	:	NM_000874	Interferon (alpha, beta and omega) receptor 2	TMEM173	:	NM_198282	Transmembrane protein 173
IFNB1	:	NM_002176	Interferon, beta 1, fibroblast	TNFSF10	:	NM_003810	Tumor necrosis factor (ligand) superfamily, member 10
IFNE	:	NM_176891	Interferon, epsilon	TRAF3	:	NM_003300	TNF receptor-associated factor 3
IFNW1	:	NM_002177	Interferon, omega 1	TYK2	:	NM_003331	Tyrosine kinase 2
				VEGFA	:	NM_003376	Vascular endothelial growth factor A

### 2.5. Viral infections

The HEK293 cells were cultured in a 12-well plate (5 × 105 cells/well) containing DMEM (+) and incubated at 37 °C in a humidified incubator with 5% CO2 for 24 h. The cells were infected with influenza A viruses at a dose of 2.0 multiplicity of infection (MOI). Before infection, the cells were washed with OPTI-MEM and the viruses diluted in 1% BSA solution were added to each well. After 30 min of incubation at 37 °C, the inoculums were completely removed. Two mL of maintenance medium (u1db8) was added to each well and incubated at 37 °C with 5% CO_2_ for 8 h. After incubation, the cells were harvested for RNA extraction. 

### 2.6. Plasmid DNA transfection

HEK293 cells were cultured in a 12-well plate (5 × 105 cells/well) containing DMEM (+) and incubated at 37 °C with 5% CO_2_ for 24 h. The cells grown in each well were transfected with 2 µg of total plasmid DNA. Plasmid DNA was diluted in OPTI-MEM at 20 ng/µL, mixed with 40 ng/µL polyethylenimine (PEI) solution and incubated at room temperature for 10 min. Then, DNA/PEI mixtures were added to the cultures. The cultures were incubated for 24 h and harvested for RNA extraction.

### 2.7. Total RNA extraction and reverse transcription

Total RNA was extracted from the virus-infected or transfected cells with the RNeasy plus mini kit (Qiagen GmbH). cDNAs were prepared from 500 ng total RNA using the Moloney murine leukemia virus reverse transcriptase (New England Biolabs Ltd.) and oligo (dT) as primer for 60 min at 45 °C.

### 2.8. Quantification real-time PCR

The quantitation of interferon-related and housekeeping gene transcripts in the cells infected with viruses or transfected was carried out with a quantitative real-time polymerase chain reaction (qRT-PCR). Total RNA and cDNAs were prepared as described above. A qRT-PCR was conducted using the FastStart Universal SYBR Green Master Mix (Roche Diagnostics GmbH, Mannheim, Germany). Equal volumes of diluted cDNA were mixed with × 2 SYBR Green Master Mix and applied to PCR amplification. The cycle conditions were applied as an initial denaturation step at 95 °C for 10 min, followed by 45 cycles of amplification for 15 s at 95 °C and 1 min at 60 °C. The quantities of the transcripts were normalized by the amount of STAT3 (signal transducer and activator of transcription 3) transcript.

### 2.9. Reporter SEAP assay

The SEAP reporter gene cloned under the control of the SV40 promoter was used to detect the effects of influenza A virus PA proteins on transcription and/or translation in HEK293 cells. The HEK293 cells were cultured in a 24-well plate (1 × 10^5^ cells/well) containing DMEM (+) and incubated at 37 °C with 5% CO_2_ for 24 h. The cells were cotransfected with a certain amount of pSEAP plasmid and increasing amounts of plasmid (pCAGGS-PA/W or pCAGGS-PA/D) encoding viral PA protein as described above. The secreted alkaline phosphatase enzyme (SEAP) activity in the medium was determined with a commercial kit (Roche Diagnostics GmbH; # 31420) 36 h after transfection following the instructions in the manual. The enzyme activity was measured as luminesces in a luminometer (GloMax 20/20 Luminometer; Promega Corp., Madison, WI, USA).

### 2.10. Western blotting

To evaluate the effects of influenza PA proteins on transcription and/or translation, plasmids encoding PA and human beta actin protein (pCHA-ACTB) were cotransfected into HEK293 cells and then endogenous actin beta and HA-tagged actin beta proteins encoded from the plasmid DNA were compared by Western blotting. Thus, HEK293 cells were cultured in a 12-well plate (2.5 × 10^5^ cells/well) for 24 h and cotransfected with pCHA-ACTB (0.75 µg/well) and PA coding plasmids (0.75 µg/well) as described above. After 48 h transfection, the cells were harvested in an RIPA buffer. The proteins in the cell lysates were separated with SDS-PAGE and transferred to the polyvinylidene difluoride (PVDF) membrane. After blocking, the membrane was first treated with mouse monoclonal anti-HA (Santa Cruz Biotechnology Inc., Santa Cruz, CA, USA; # sc-7392) or anti actin (MyBioSource, Inc., San Diego, CA, USA; # MBS9400413) followed by horseradish peroxidase-conjugated second antibody [antimouse IgG-HRP (Invitrogen Corp., Carlsbad, CA, USA; # 31420). The actin proteins were visualized with an ECL detection kit (GE Healthcare Srl, Milan, Italy).

### 2.11. Statistical analysis

The statistical significance of differences between experimental groups was evaluated using analysis of variance (one-way ANOVA with Newman–Keuls post test) in the SPSS. P values less than 0.05 were considered statistically significant.

## 3. Results

### 3.1. Quantitation of interferon-related gene transcripts with PCR-array in infected cells

**Figure 1:**
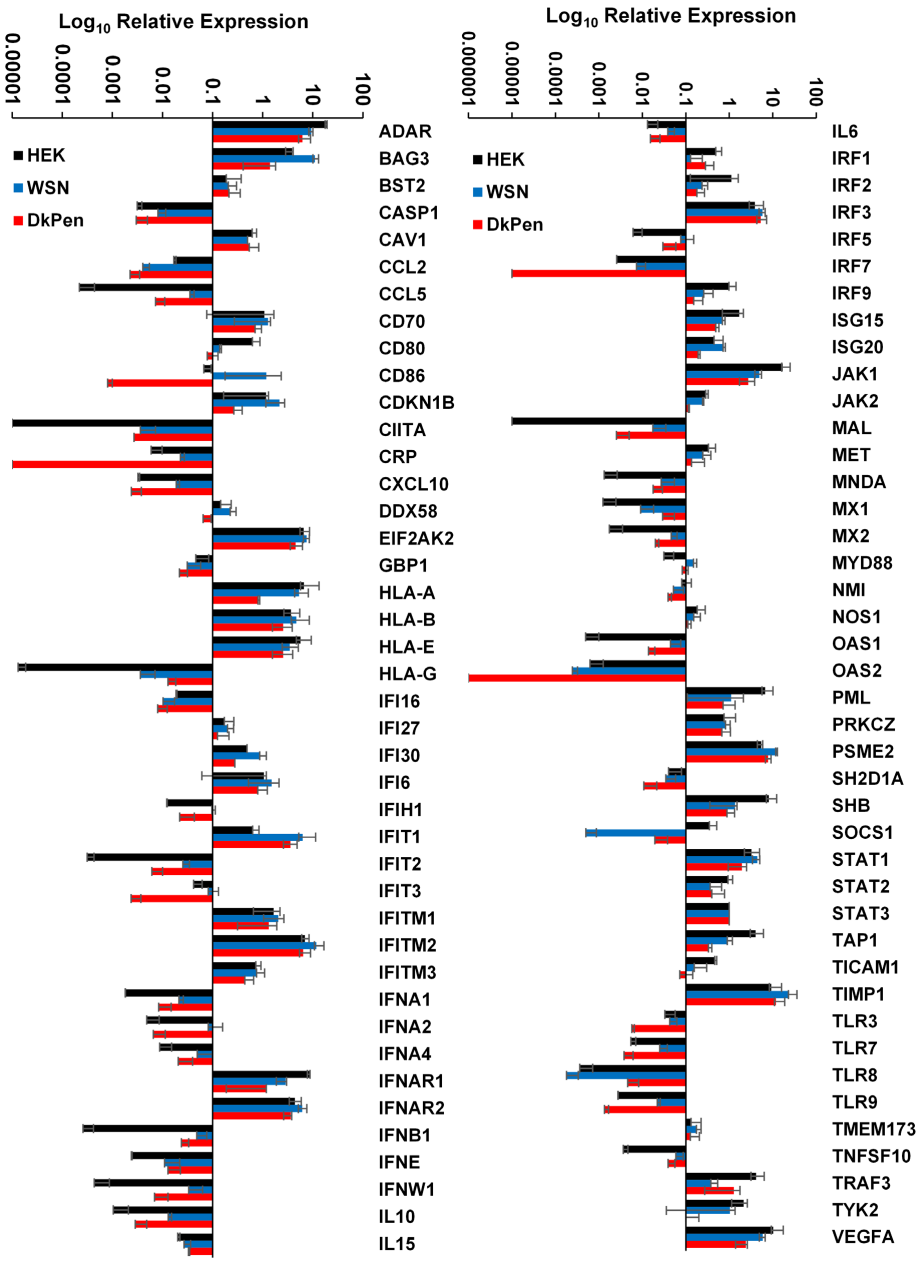
The expression profiles of interferon response genes in HEK293 cells infected with influenza A viruses (WSN or DkPen) and noninfected cells (HEK293).

**Figure 2:**
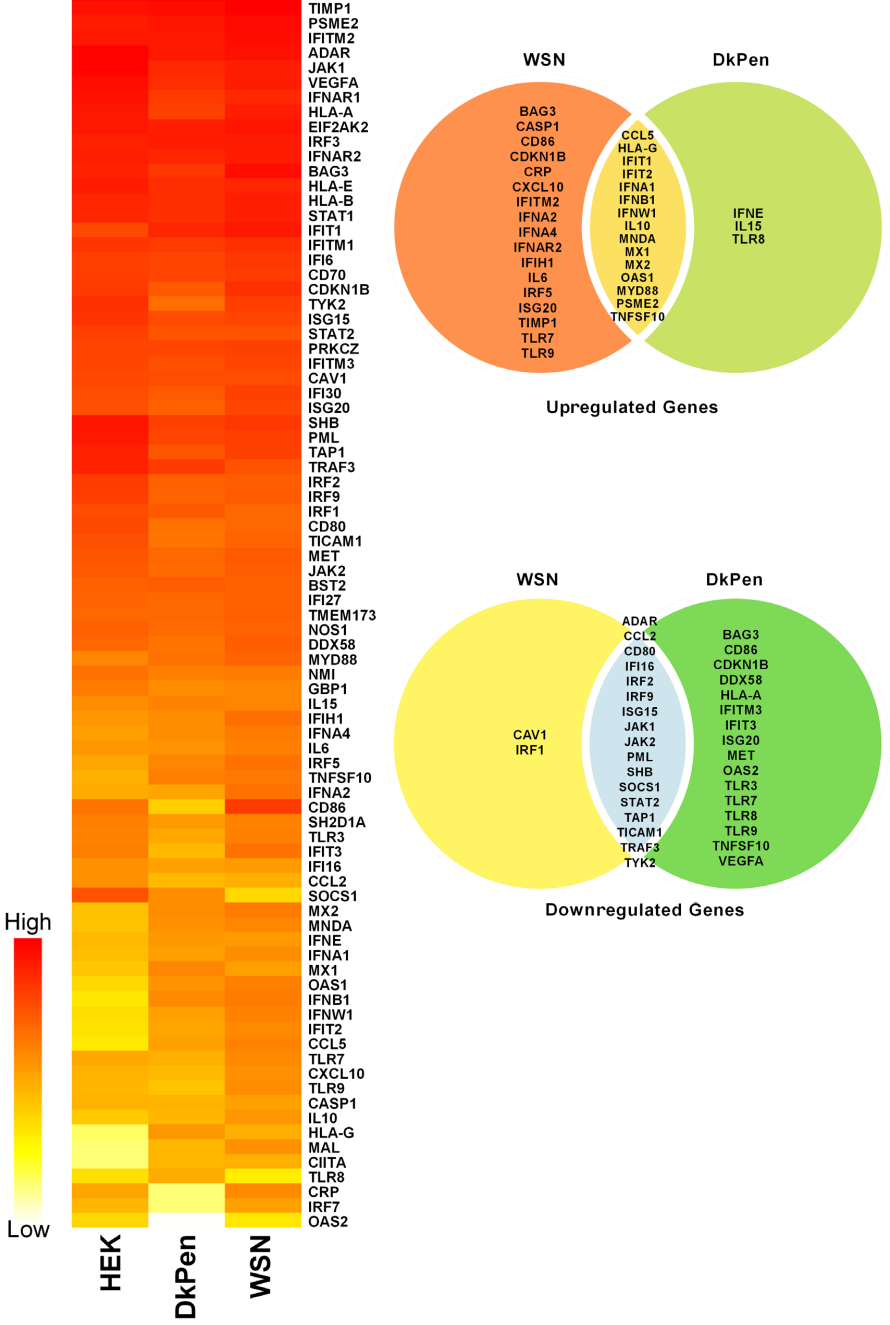
The heatmaps and Venn diagram of the genes related to the interferon response in HEK293 cells infected with influenza A viruses (WSN or DkPen) and noninfected cells (HEK293).

Viruses carrying the RNA genome, such as influenza A viruses, often induce interferon synthesis and activate the interferon pathway and expression of related genes. Therefore, several proteins related to interferon response have a relationship with influenza A virus proteins and interfere with viral replication (u1db9; u1dba). In this study, the transcript levels of the genes related to interferon responses in HEK293 cells infected with two different types of influenza A virus or transfected with plasmids coding viral RNA polymerase enzyme subunits were analyzed by quantitative real-time PCR techniques. The assay was carried out with the plates designated for interferons and interferon related genes in a 96-well format (Qiagen 96-well / RT² Profiler PCR Array (PAHS-016ZD-6). The transcripts of 84 interferon-related genes and five different housekeeping genes (ACTB, B2M, GAPDH, HPRT1, and RPLP0) were quantified in the cDNAs prepared from virus-infected cells. The assay was repeated at least three times for each group. The relative quantity (ΔCq) values of each transcript were normalized with STAT3 transcript, which varies relatively less in infection conditions and moderate levels, and the results were evaluated. The results are given both in bar graphs and in a heatmap (Figures 1 and 2). 

As a result of statistical evaluations, 32 genes were found to be upregulated in the cells infected with human-type influenza WSN virus compared to uninfected cells. Among these gene transcripts, the P value was determined as ≤ 0.01 for 18 genes and 0.05–0.01 for 14. The transcript level of 20 genes was downregulated in infected cells (P value ≤ 0.01 for 13 genes; 0.05–0.01 for 6 genes). It was not observed that important changes in the transcript level of other genes related to the interferon pathway (Figure 2). It was found that the most increased transcripts in the cells infected with WSN viruses were encoded by the genes CCL5 (160 fold), HLA-G (275 fold), IFIT2 (81 fold), IFNB1 (185 fold), IFNW1 (76 fold) and OAS1 (89 fold). The most decreased transcript in the cells infected with this virus was the SOCS1 gene transcript (697 fold).

The primary host organisms of influenza A viruses are wildfowl, especially aquatic birds. Although virus transmission between different species is not easy, influenza viruses can cross species barriers and infect humans from time to time. Depending on both cellular and viral factors, avian influenza A viruses replicate much more slowly in mammalian cells (u1dbb). Most of the cellular factors causing this situation are unknown. In this study, the transcript levels of genes related to the interferon pathway were investigated not only in cells infected with human type influenza WSN viruses but also in cells infected with avian type influenza DkPen. It was shown that 18 genes were upregulated (P value ≤ 0.01 for 12 genes; 0.05–0.01 for 6 genes), 32 genes were downregulated (P value ≤ 0.01 for 26 genes; 0.05–0.01 for 6 genes) and the transcript levels of the remaining genes were not significantly changed in the cells infected with DkPen viruses. Among the genes with significantly differing transcript levels between virus-infected and noninfected cells, the highest increase values were detected in the transcripts of CCL5 (33 fold), HLA-G (980 fold), IFNB1 (92 fold) and OAS1 (28 fold). The genes with the most decreased transcript levels were OAS2 (627 fold) and SOCS1 (18 fold). If an assessment is made between the transcript levels of genes quantified in influenza WSN and DkPen infected cells, it appears that more interferon-related genes were downregulated in DkPen infected cells (DkPen infected/32 genes; WSN infected/19 genes), while fewer genes were upregulated (DkPen infected/18 genes; WSN infected/32 genes) (Figure 2). 

**Figure 3:**
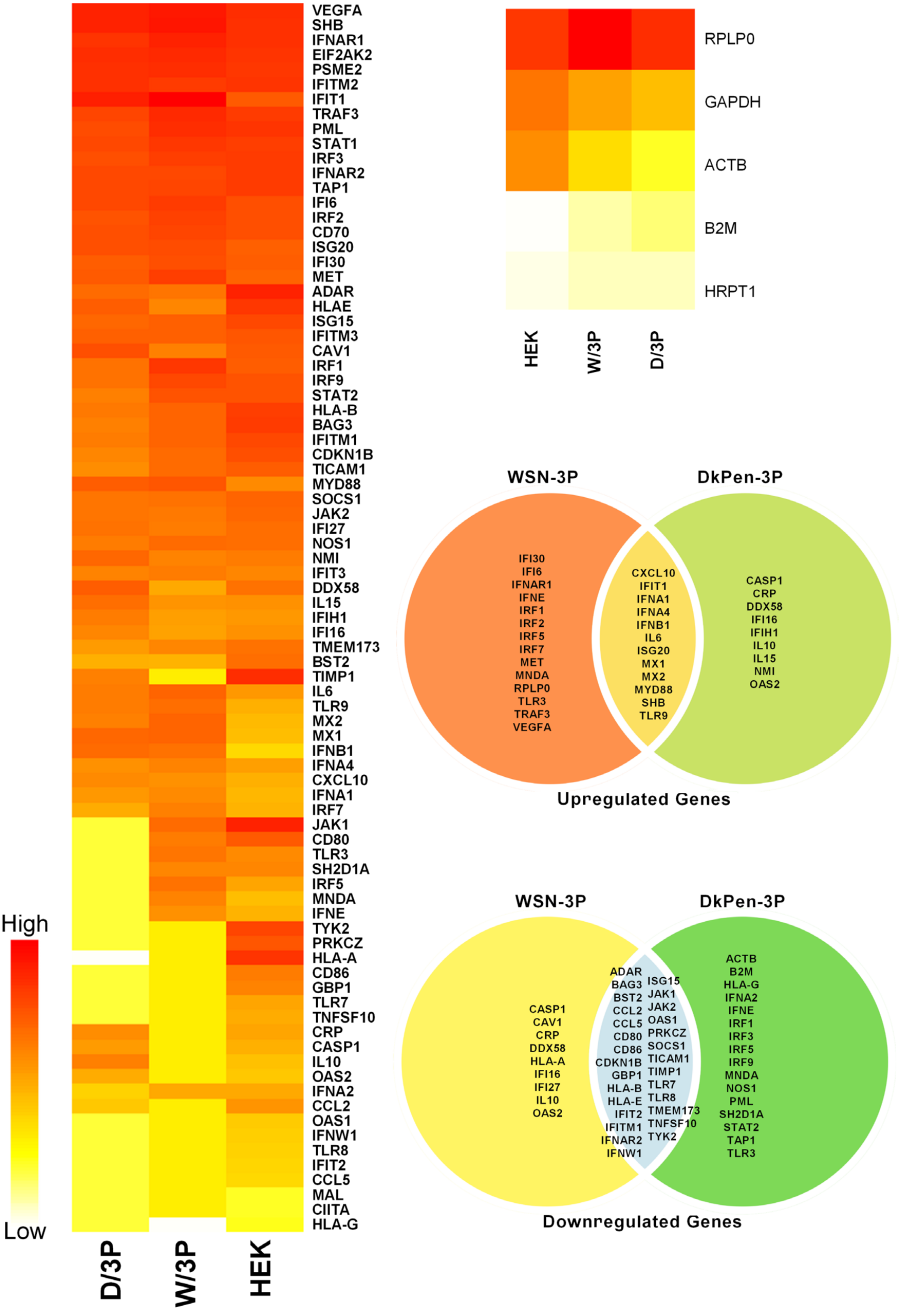
The heatmaps and Venn diagram of the interferon response genes in HEK293 cells transiently transected with plasmid DNAs expressing influenza A virus RdRP enzyme (3P).

The housekeeping genes were evaluated in a separate category since they were not associated with interferons. The relative transcript quantities of ACTB, B2M, GAPDH, HPRT1, and RPLP0 genes in uninfected cells and in the cells infected with viruses are given in Figure 3. 

The results show a statistically significant (P = 0.0001) average 5.5-fold decrease of ACTB gene transcript levels in the cells infected with influenza DkPen viruses compared to uninfected cells. No important changes were detected in other housekeeping gene transcript levels in DkPen infected cells. In cells infected with influenza WSN viruses, the ACTB gene transcript level was also decreased (P = 0.0426), but not as much as in the cells infected with DkPen viruses. Only a 1.16-fold reduction of ACTB transcript was detected in WSN infected cells. A relative increase in the transcript level of the other housekeeping genes was observed in the cells infected with influenza WSN viruses (Table 2). 

**Table 2:** The fold changes in housekeeping gene transcript levels in HEK293 cells infected with influenza A viruses compared to control cells.

GenBank	Symbol	Gene name	P value	Fold change HEK/WSN	P value	Fold change HEK/DkPen
NM_001101	ACTB	Actin, beta	0.0426	1.16	0.0001	5.55
NM_004048	B2M	Beta-2-microglobulin	0.0001	0.40	0.0480	0.76
NM_002046	GAPDH	Glyceraldehyde-3-phosphate dehydrogenase	0,0179	0.65	0.5220	1.11
NM_000194	HPRT1	Hypoxanthine phosphoribosyltransferase 1	0.0001	0.58	0.4900	0.90
NM_001002	RPLP0	Ribosomal protein, large, P0	0.02	0.78	0.8270	0.98

### 3.2. Quantitation of interferon-related gene transcripts with PCR-array in transfected cells

**Figure 4:**
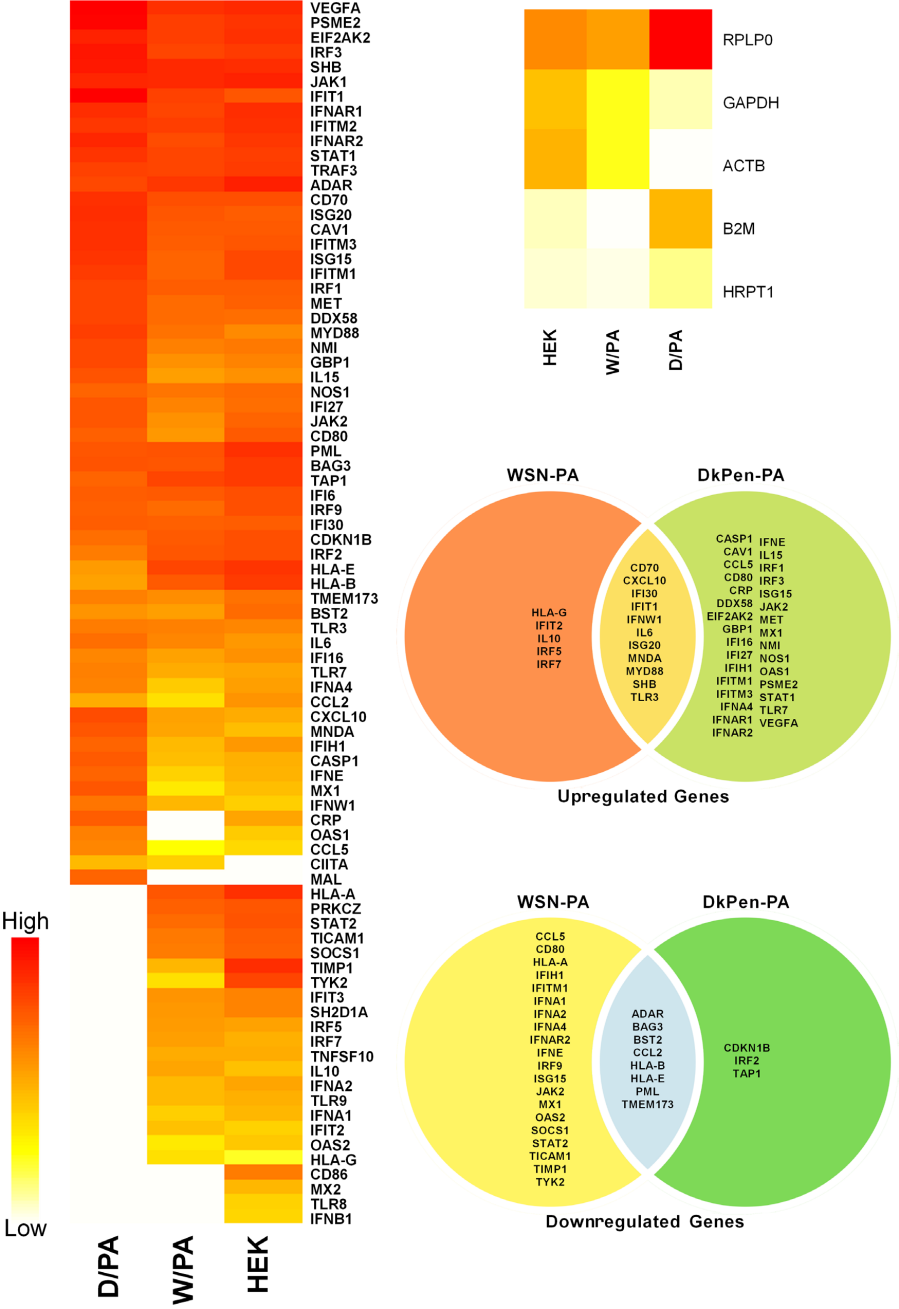
The heatmaps and Venn diagram of the interferon response genes in HEK293 cells transiently transected with plasmid DNAs expressing the PA subunit of influenza A virus RdRP.

It has been shown in previous reports that influenza A virus RdRP enzyme subunits interact with many cellular proteins and some of these proteins are related to the interferon defense system (u1dbc; u1dbd). Based on the reports, the transcript levels of interferon response genes in HEK293 cells transiently transfected with plasmids (pGAGGS-PB2, pGAGGS-PB1, pGAGGS-PA) encoding influenza A virus RdRP enzyme or only PA subunit (pGAGGS-PA, pGAGGS-PA/D-W or pGAGGS-PA/W-D) were investigated. It has been shown that the transcript levels of the genes in HEK293 cells expressing three subunits (3P) of the viral RdRP enzyme show some differences in expression profiles to those infected with the viruses. The reason is thought to be the synthesis of other viral proteins in infection conditions. However, considering the gene expression profiles of the cells expressing WSN and DkPen RdRP subunits in general, fewer genes were upregulated in the cells expressing the DkPen RdRP enzyme such as in infected cells while more genes were downregulated (Figure 3). On the other hand, significant differences in the expression profiles of interferon response genes were also shown in the cell expression PA protein alone (Figure 4). In particular, avian type influenza PA protein was found to cause more aggressive changes on the transcript levels of the genes. This result shows that influenza A virus PA protein is one of the important viral factors affecting cellular transcription.

### 3.3. The effects of influenza A virus PA proteins on Pol II transcripts in HEK293 

**Figure 5:**
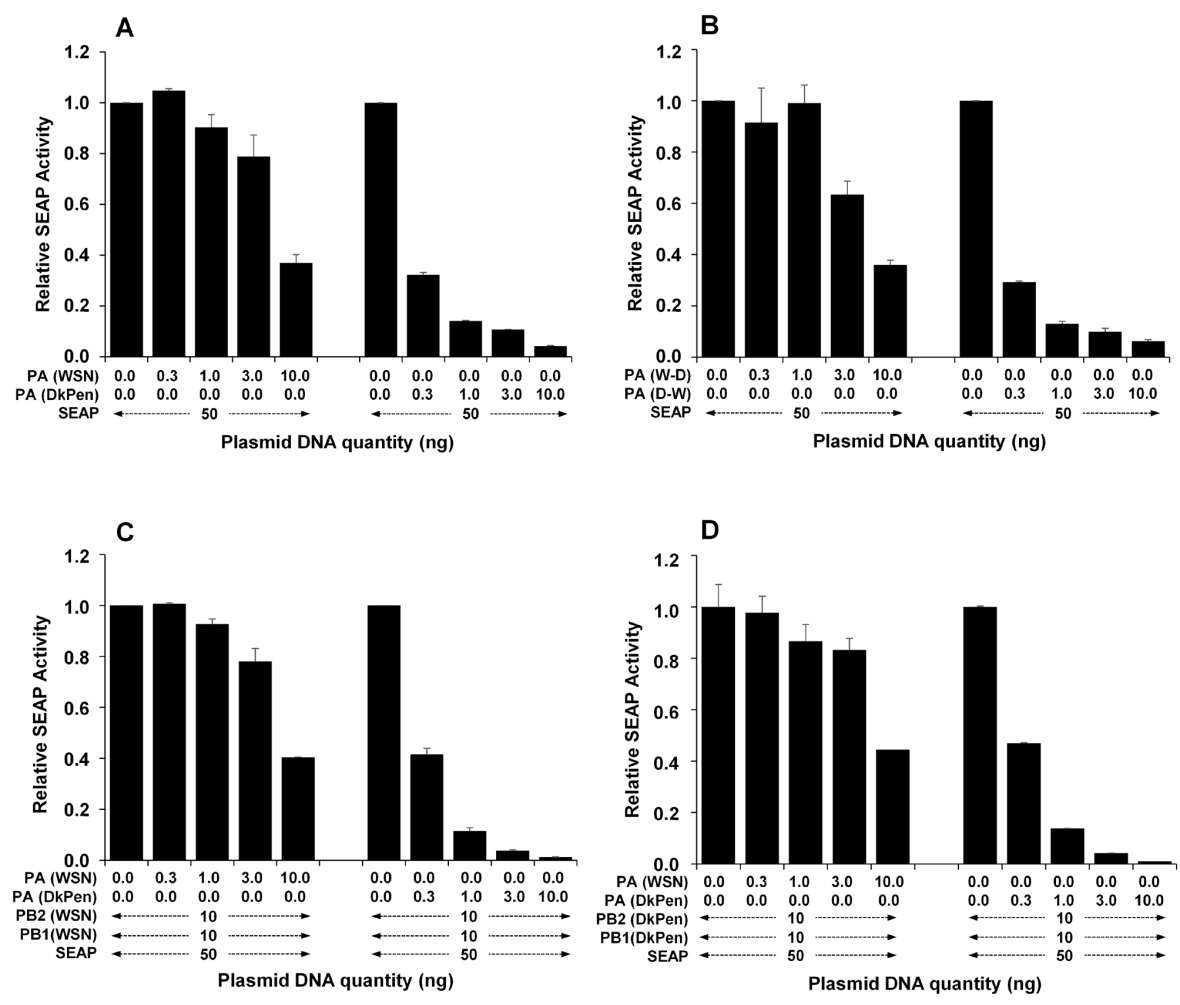
The effects of influenza A virus PA proteins on the expression of SEAP reporter in transiently transfected HEK293 cells. A. The cells expressing native PA proteins. B. The cells expressing chimeric PA proteins. C. The cells expressing influenza PA proteins with WSN PB2 and PB1. D. The cells expressing influenza PA proteins with DkPen, PB2 and PB1. Total plasmid DNA adjusted to 250 ng/well with pCAGGS plasmid DNA (Niwa et al., 1991).

**Figure 6:**
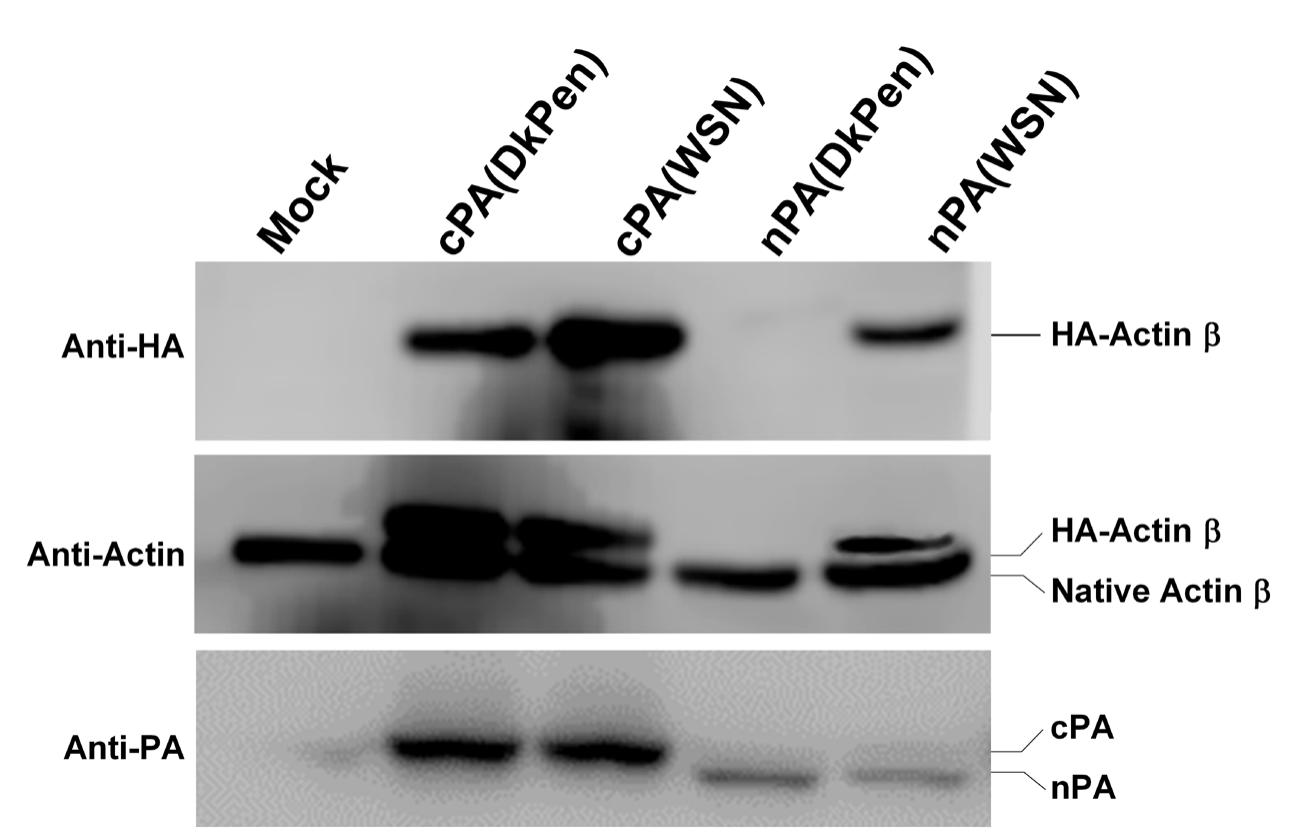
Western blot analysis of native actin beta and HA-actin proteins in transiently transfected HEK293 cells. The cells were cotransfected with pCHA-ACTB plasmid and a plasmid expressing deleted PA protein (pCAGGS-cPA/DkPen, pCAGGS-cPA/WSN, pCAGGS-nPA/DkPen or pCAGGS-nPA/WSN). Actin beta and viral PA proteins were separated on 10% polyacrylamide gel and immunoblotted with monoclonal mouse anti-HA (for HA-ACTB), monoclonal anti actin (for ACTB and HA-ACTB) and rabbit polyclonal anti-PA antibodies.

The effects of the PA subunit of influenza A virus RdRP enzyme causing aggressive changes in the cellular transcripts were investigated on the synthesis of reporter-secreted alkaline phosphatase (SEAP) enzyme and human ACTB protein encoded from the plasmid DNAs in HEK293 cells. For this purpose, a certain amount of pSEAP plasmid DNA was cotransfected with increasing amounts of plasmid DNA encoding of influenza A virus PA protein (pCAGGS-PA/WSN, pCAGGS-PA/DkPen, pCAGGS-PA/D-W or pCAGGS-PA/W-D) into the cells. After 36 h of transfection, reporter SEAP activities secreted into the medium were detected and the results are given in Figure 5. As shown in Figure 5A, the PA protein of influenza DkPen more efficiently blocked the reporter SEAP expression compared to influenza WSN PA protein in a dose-response manner. The chimeric PA (D-W) protein consisting of the amino terminal half of DkPen PA and the carboxy terminal half of WSN PA, encoded from pCAGGS-PA/D-W plasmid, blocked SEAP expression such as DkPen PA protein, while the PA(W-D) (encoded from the pCAGGS-PA/W-D) behaved like WSN PA protein (Figure 5B). This result revealed that the amino terminal half of the DkPen PA was responsible for the inhibition of SEAP expression in HEK293 cells. To determine if the synthesis of PA proteins with the other subunits of the viral RdRP (PB2 and PB1) affects the result, the cells were cotransfected with plasmids encoding these proteins. The results show that the negative regulatory effect of viral PA protein on the gene expression was independent of the other subunits of the viral RdRP enzyme. (Figures 5C and 5D). The negative regulatory effects of PA proteins, especially amino terminal halves, on the gene expression were also investigated by Western blotting of actin protein tagged with HA encoded from plasmid DNA and endogenous actin beta. For this purpose, HEK293 cells were co-transfected with the pCHA-ACTB plasmid and plasmids encoding the amino and carboxy terminal halves of the WSN and DkPen virus PA proteins. After 48 h transfection, the cells were lysed in SDS-PAGE sample loading buffer and both endogenous actin and HA-tagged actin proteins were analyzed with Western blotting (Figure 6). The results revealed that the synthesis of HA-tagged actin encoded from the plasmid DNA was completely inhibited by the DkPen nPA protein, while there was no important reduction in the amount of endogenous actin beta protein. The WSN nPA protein showed lower negative effects on the actin beta synthesis from the plasmid DNA, whereas both WSN cPA and DkPen cPA proteins had no inhibitory effects on the actin gene expression.

## 4. Discussion

Viruses with the RNA genome in different families cause important changes in the expression profile of many host genes during their replication processes (u1dbe). This is due to the predominant use of cellular metabolites such as nucleoside triphosphates in the synthesis of virus components and/or the transcriptional and posttranscriptional regulation of the host gene expression with various viral proteins (u1dbf). In this work, the expression profiles of a limited number of genes related to interferon response in the human originated cells infected with influenza A viruses or transfected with plasmid DNAs encoding the viral RdRP enzyme subunits were investigated by the PCR array technique. The expression profiles of 84 interferon response genes and five housekeeping genes in the cells infected with human type influenza WSN and avian type influenza DkPen virus were analyzed. The data obtained as a result of normalization with STAT3 gene expression level showed that 32 genes in WSN virus-infected cells and 18 genes in DkPen virus-infected cells were upregulated. Among these genes, 15 were found to be upregulated in both cells infected with two different types of virus (Figure 2). In contrast, 19 genes in WSN-infected cells and 32 genes in DkPen-infected cells were determined to be downregulated and 17 genes were common to both cells. These gene expression profiles resulted in the conclusion that the influenza DkPen viruses negatively regulate the expression of more genes in the host cells than those of WSN viruses. Although no experimental work to explain the mechanism of action on virus replication was carried out, CCL5, HLA-G, IFNB1, IFNW1, OAS1, and IFIT2 genes were found to be prominent in terms of high expression levels in infected cells. The highest expression of these genes in both types of virus-infected cells suggested that they have great importance in viral replication, regardless of the virus type. Genes whose transcript levels decreased most in the virus-infected cells were determined to be SOCS1 and OAS2. The SOCS1 gene was found to be downregulated in both types of virus-infected cells, while the OAS2 gene decreased only in the cells infected with DkPen viruses.

Human leukocyte antigen G (HLA-G) is known as an important host genetic susceptibility factor for human immunodeficiency virus (HIV), human papillomavirus (HPV), human cytomegalovirus virus (HCMV), and even for the vertical transmission of HIV infections in humans. It has been found that both the soluble and the membrane-bound HLA-G levels increase in virus-infected patients or cells (u1dc0). An increase in the level of HLA-G has also been demonstrated in patients infected with influenza A viruses (u1dc1). In this study, it was shown that the HLA-G gene expression was highly upregulated in the HEK293 cells with both human and avian type influenza A virus infections. It was reported that the CCL5 gene, which was found to be upregulated in the HEK293 cells with influenza A virus infection in this work, negatively regulates virus replication in mice and that mice having a mutated CCL5 gene are very sensitive to influenza virus infections (u1dc2). The OAS1 gene induced with interferons encodes 2’-5’-oligoadenylate synthetase 1 enzyme has antiviral properties (u1dc3). This gene was also found among the highly upregulated genes in cells infected with both influenza WSN and DkPen viruses. IFNB1 and IFNW1 genes, which encode interferon beta and interferon omega-1 proteins, respectively, are among the genes with the highest increase in transcript levels. The SOCS1 (SSI-1) gene, which is among the genes related to interferon response and is significantly downregulated in the virus-infected cells, encodes the suppressor of cytokine signaling 1 protein (u1dc4). It has been reported that SOCS1 and SOCS3 proteins act as negative regulators for the lung mucosal innate immune response due to influenza A virus infection (u1dc5). Here, a significant decrease in SOCS1 transcript levels in the cells infected with both WSN and DkPen influenza A virus was detected. However, there is not much data showing the importance of SOCS1 downregulation for influenza A virus infections. The changes in gene expression of the host cells during viral infection do not occur only at the transcription stage. Some viral proteins also lead to destabilization or degradation of host cell transcripts. Influenza A virus NS1 protein blocks the polyadenylation of the host mRNAs and causes it to be destabilized while the polyadenylation of viral mRNAs is performed with the viral RdRP enzyme and is not affected by this situation (u1dc6). Another important effect of the NS1 protein is on the splicing of host mRNAs. This protein binds to specific regions of human U6 snRNA and prevents interaction of U6-U2 and U6-U4 snRNA for splicing (u1dc7). One of the most important viral factors affecting the stability of host cell transcripts is the influenza virus RdRP enzyme. The RdRP enzyme cuts the cap structures of newly synthesized mRNAs in the cells with cap-snatching activity and uses them as a primer for the synthesis of viral mRNAs (u1dc8). Consequently, RdRP is an especially important viral factor leading to the destabilization of cellular transcripts. Recent studies have revealed that the PA subunit of the RdRP enzyme has nuclease activity and an important role in the cap-snatching process (u1dc9; u1dca). Consequently, in this study, interferon response gene transcript levels were also analyzed in the HEK293 cells synthesizing the viral RdRP enzyme (PB2, PB1, and PA) or only the PA subunit (Figures 3 and 4). The results show that the gene transcription profiles in the cells that synthesize three polymerase subunits (PB2, PB1, and PA) as a complex were closer to the transcript profiles of the virus-infected cells. In our previous study (Pham et al., 2018), the PA component of the viral RdRP composed of different combinations of WSN and/or DkPen polymerase subunits was found the most determinative subunit for enzyme activity in mammalian HEK293 cells. Therefore, the effects of PA protein on host cell transcript level in HEK293 cells were also evaluated. In contrast to viral RdRP complex, more aggressive changes at the gene transcript level were observed in the cells expressing the PA subunit alone compared to control HEK293 cells. It has been shown that the DkPen virus PA protein in particular causes a noticeable upregulation of some genes while leading to a severe decrease in other gene transcripts (Figure 4). Although we have insufficient proof, PA proteins are thought to play an important role in the destabilization of host gene transcripts, especially those that are newly synthesized. In order to support this idea, the effects of native or mutated PA proteins on host RNA polymerase-dependent transcription have been indirectly investigated by detection of the reporter SEAP enzyme activity and the actin beta protein encoded from the plasmid vectors. The reporter SEAP enzyme synthesis was inhibited with PA proteins in a dose-dependent manner when co-synthesis of these two proteins in transfected HEK293 cells (Figure 5). However, when PB2 or PB1 protein was synthesized alone, no significant reduction was observed in SEAP activity (data not shown). Avian type influenza DkPen virus PA protein was found to inhibit SEAP expression to a much higher degree than that of influenza WSN PA protein and this inhibition was independent of other viral RdRP subunits. The results obtained with chimeric PA proteins reveal that the PA(D-W) protein, consisting of the amino-terminal moiety of DkPen PA, has a high inhibition on reporter SEAP gene expression. It is known from previous studies that the amino terminal domain of influenza PA protein has endonuclease activity (u1dcb). Therefore, the results suggest that DkPen PA protein has much higher endonuclease activity than that of WSN PA protein. It has been shown that deleted PA protein, which consists of the amino terminal half of DkPen PA, almost completely inhibits the synthesis of actin beta protein tagged with HA encoded from plasmid DNA (Figure 6). This result supports the hypothesis that DkPen PA has higher endonuclease activity than that of WSN PA in the cells. Although the cap-snatching activity assay performed under in vitro conditions shows that WSN virus PA protein has higher activity (u1dcc), it is thought that this result may be different in natural conditions within the cells.

While influencing host preferences of influenza A viruses are the factors that affect the HA protein of the virus and the receptor proteins that this protein attaches to the cells, other protein factors affect the progression of viral replication in the cell. The main factors for the host preferences and pathogenesis of influenza A viruses are the HA protein of the virus and the receptor proteins that this protein attaches to the cells. However, there are other viral protein factors such as viral RdRP enzyme in the cells that affect the progression of viral replication (u1dcd). Different types of influenza A virus RdRP enzymes have different levels of activity in mammalian cells (u1dce). The data obtained from this study suggest that one of the important factors limiting the replication of avian viruses in mammalian cells is the viral RdRP enzyme. In particular, the viral RdRP enzyme PA subunit is thought to have a negative effect on cellular gene expression and replication itself.

In this study, human HEK293 cells were used to determine the effects of influenza virus infections on interferon-related gene expression in mammalian cells. It has been shown that nonadaptive avian type influenza viruses encounter resistance of various host factors within the cell during replication in mammalian cells (Beare and Webster, 1991; Moncorgé et al., 2010). Therefore, it is expected that human and avian type viruses have different effects on gene expression profiles associated with interferons in HEK293 cells. On the other hand, it has been a matter of curiosity how especially avian type DkPen viruses will affect the expression of interferon related genes in chicken cells. The absence of PCR microarray kit developed for chicken cells, equant to mammalian interferon related gene set (RT² Profiler PCR Array), did not allow working with these cells. However, the fact that the avian type viruses can replicate more easily in chicken cells, it is thought that influenza A viruses will differently affect interferon-related gene expression profiles in these cells than the genes of mammalian cells.

In summary, in this study, the transcript levels of interferon response genes in HEK293 cells infected with two different influenza A virus types or transiently transfected with plasmid DNAs encoding the viral RdRP subunits were analyzed by the PCR array technique. It has been revealed that some interferon response genes give similar expression profiles in HEK293 cells infected with both human and avian influenza A viruses while others show opposite expression profiles. A higher number of interferon-related genes were found to be downregulated in the cells infected with avian type influenza DkPen viruses compared to the human type A WSN. In particular, it was concluded that the PA protein, a subunit of viral RdRP enzyme, is one of the important viral factors affecting the expression profiles of host interferon response genes.
